# Giant Cell Tumor within the Proximal Tibia after ACL Reconstruction

**DOI:** 10.1155/2016/2820381

**Published:** 2016-02-14

**Authors:** Takashi Takahashi, Lauren MacCormick, Jutta Ellermann, Denis Clohisy, Shelly Marette

**Affiliations:** ^1^Department of Radiology, University of Minnesota Medical Center, 420 Delaware Street SE, MMC 292, Minneapolis, MN 55455, USA; ^2^Department of Orthopaedic Surgery, University of Minnesota Medical Center, 2450 Riverside Avenue, Suite R200, Minneapolis, MN 55454, USA

## Abstract

26-year-old female with prior anterior cruciate ligament reconstruction developed an enlarging lytic bone lesion around the tibial screw with sequential imaging over the course of one year demonstrating progression of this finding, which was confirmed histologically to be a giant cell tumor of bone. The lesion originated around the postoperative bed, making the diagnosis challenging during the early course of the presentation. The case demonstrates giant cell tumor which originated in the metaphysis and subsequently grew to involve the epiphysis; therefore, early course of the disease not involving the epiphysis should not exclude this diagnosis.

## 1. Introduction

There have been only two previous reported cases of giant cell tumor around the fixation screws after bone-patella-bone anterior cruciate ligament reconstruction. This is the third reportable case where sequential images demonstrate development of a new giant cell tumor within the proximal tibia around the tibial screw. Retrospectively, image findings demonstrate classic features of giant cell tumor of the bone. However, the postoperative timing as well as the initial location of the lesion in the metaphysis with subsequent extension in the epiphysis made the diagnosis challenging for experienced musculoskeletal radiologists, as well as orthopedic surgeons in the early disease course. The case demonstrates and confirms the origin of the tumor in the metaphyseal region as previously suggested.

## 2. Case Report

**Figure 1:**
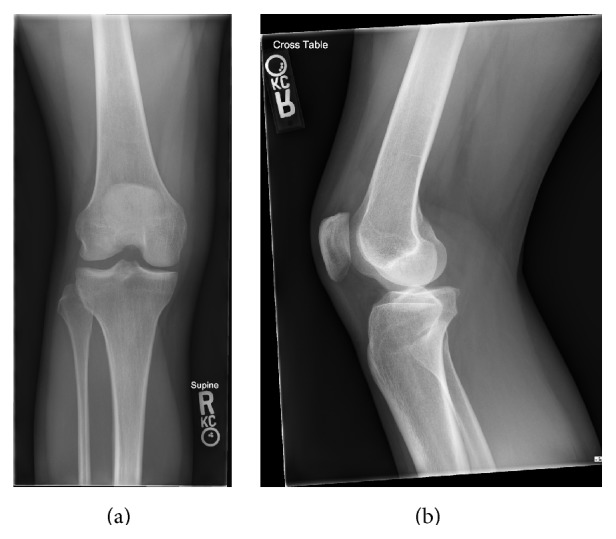
AP (a) and lateral (b) view radiographs of right knee for the evaluation of acute injury. Lateral view demonstrates moderate joint effusion, deepening of the terminal sulcus, and irregularity of the posterior aspect of the lateral tibial plateau.

**Figure 2:**
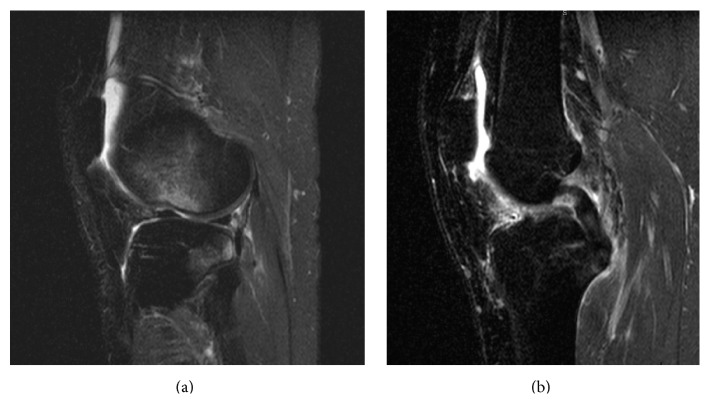
T2 weighted fat suppressed sagittal plane MRI images for evaluation of acute knee injury. Classic marrow contusion pattern at the lateral terminal sulcus and posterior aspect of the lateral tibial plateau (a) and complete rupture of anterior cruciate ligament (b).

A 26-year-old female sustained an injury to her right knee at a dance tryout. Radiographs and MRI were obtained for the evaluation of the acute injury (Figures 1 and 2). Imaging demonstrated a complete tear of her anterior cruciate ligament, terminal sulcus impaction injury, and a corner fracture of the posterior aspect of the lateral tibial plateau.

**Figure 3:**
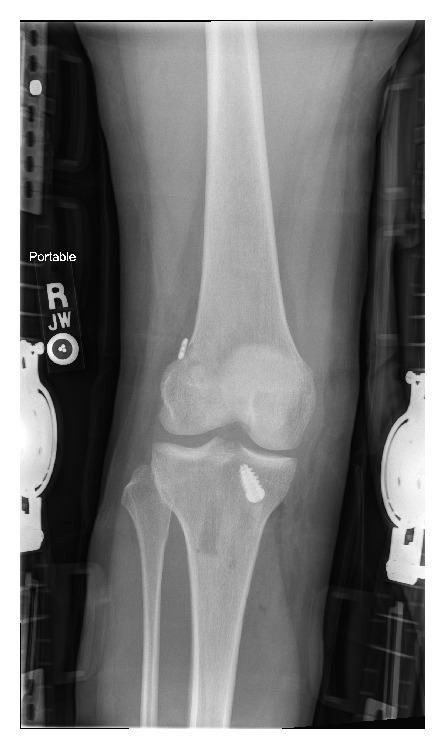
Immediate ACL reconstruction postoperative radiographs in AP view.

The patient subsequently underwent an ACL reconstruction utilizing bone-patellar tendon-bone autograft at an outside institution with an uneventful recovery. The initial postoperative radiographs from the outside institution were unremarkable (Figure 3).

**Figure 4:**
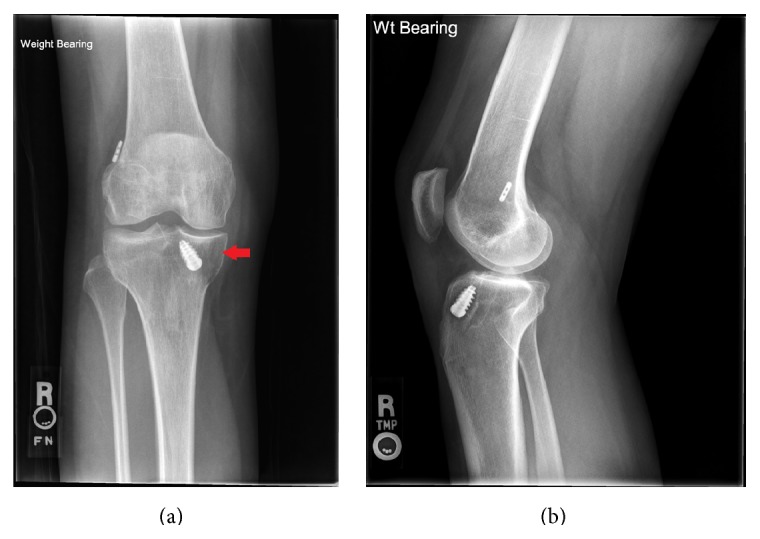
AP (a) and lateral (b) weight bearing radiographs of right knee obtained at the time of knee reinjury, approximately 6 months after the ACL reconstruction surgery. Retrospectively, new lucent lesion adjacent to the tibial tunnel is present (arrow).

**Figure 5:**
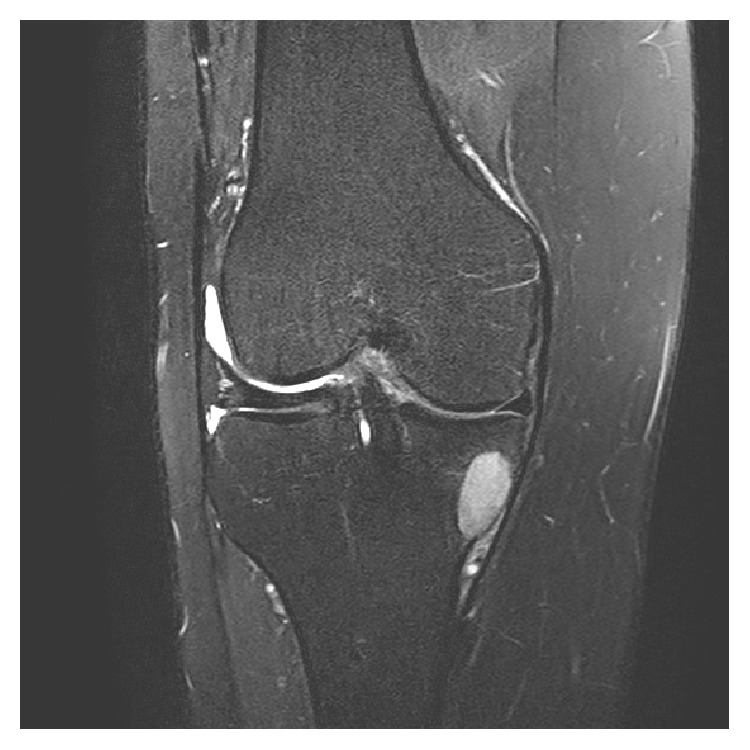
T2 weighted fat suppressed MRI in coronal plane shows well circumscribed hyperintense lesion in the medial tibial plateau without surrounding marrow edema. No communication of the lesion with tibial tunnel was present.

**Figure 6:**
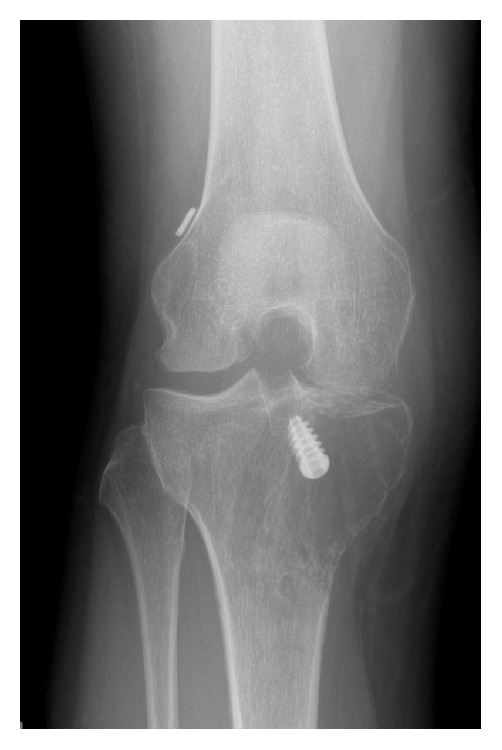
AP radiograph of the right knee shows significant progression of the tibial plateau osteolytic lesion in the medial tibial plateau, which now extends to the epiphysis.

Approximately 6 months after the ACL reconstruction surgery, the patient had reinjured her right knee from a slip and fall on the ice. Radiographs were obtained at this time (Figure 4), with the initial interpretation by an outside institution, read as being unremarkable. The patient's symptoms persisted; therefore, A MRI was obtained which demonstrated a new lesion in the anteromedial aspect of the tibia, adjacent to the tibial tunnel (Figure 5). No specific differential diagnosis was given by the outside radiologist. Additional radiographs were obtained approximately 10 months after the ACL reconstruction due to the patient's persistent symptoms. Radiographs from this time (Figure 6) demonstrated slight interval increase in the size of the medial tibial lesion. At this time, the patient was treated with steroid injection for her symptoms by the outside institution.

**Figure 7:**
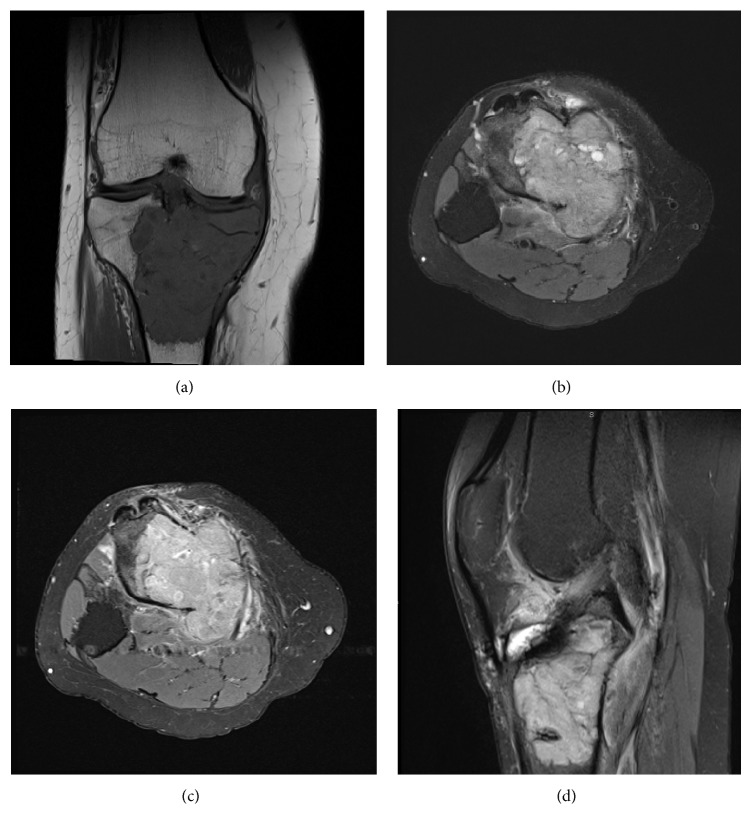
Coronal T1 weighted (a), axial fat suppressed proton density (b), and axial fat suppressed T1 postcontrast (c) images show enhancing bony lesion involving the tibial plateau with cortical breakthrough posteriorly. Selective lateral fat suppressed proton density (d) shows no evidence of other lesions in the distal femur and patella.

**Figure 8:**
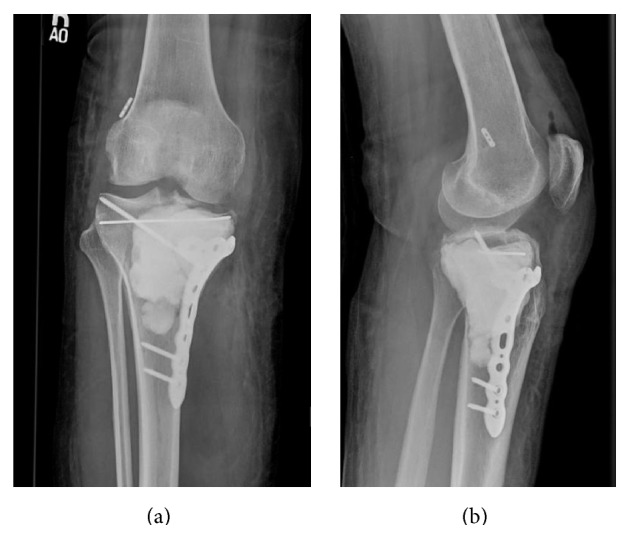
AP and lateral view radiographs immediately after curettage and packing of the proximal tibial giant cell tumor along with medial collateral ligament (MCL) repair; prophylactic fixation of the right tibia.

The patient was referred to our tertiary referral center for further evaluation of the bone lesion and associated tenderness over the anteromedial aspect of the patient's knee in the area of tibial tunnel. Review of outside films by a musculoskeletal radiologist and an orthopedic surgeon in our center included a differential diagnosis of infection, benign fibrous lesion such as fibrous dysplasia, and nonossifying fibroma. At this point, the patient was lost for further follow-up at our institution due to combination of patient's status of serving in the military and living out of state. Approximately 1 year and 7 months after the ACL reconstruction, the patient was referred back to our tertiary referral center for further assessment and treatment of the medial tibial bone lesion. Radiographs and MRI from the outside institution (Figures 6 and 7) showed significant progression of the lesion. A biopsy was recommended at this time. We discussed with the patient that the most likely diagnosis was a giant cell tumor, but it was also possible that this was a malignancy due to the aggressive nature of the tumor over the course of one year. We counseled the patient on our plan to proceed with treatment of the tumor if histology confirmed a giant cell tumor, or we would stage and plan surgical intervention at a later date if histology was consistent with a malignancy. Intraoperatively, frozen section of the lesion was consistent with giant cell tumor. At that time, we proceeded with curettage and cementation of the tumor as well as removal of prior ACL screw. Due to extension of the tumor into the medial articular surface, posterior cortex, and insertion site of the medial collateral ligament (MCL), prophylactic fixation of the right tibia and repair of the MCL with soft tissue mobilization and transfer were also performed (Figure 8). Hematoxylin and eosin stain histological slide demonstrates characteristic giant cells (Figure 11).

**Figure 9:**
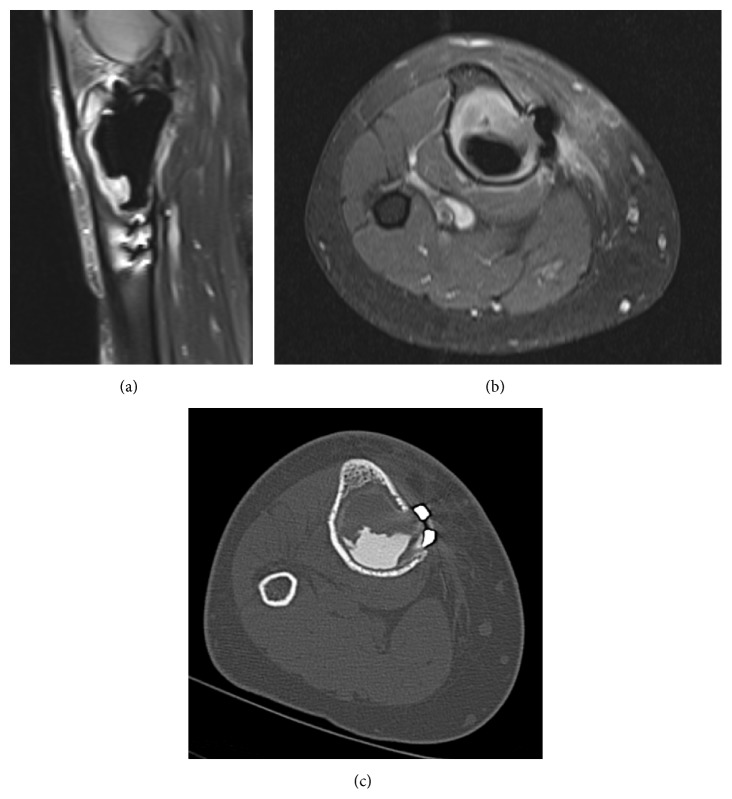
Sagittal T2 weighted fat suppressed image (a) and axial postcontrast enhanced T1 weighted image (b) of the right knee 3 months after the giant cell tumor curettage and packing demonstrate area of T2 hyperintensity and associated enhancement anterior to the surgical curettage cavity. CT scan image (c) from the same date also shows that there is corresponding uncemented area.

**Figure 10:**
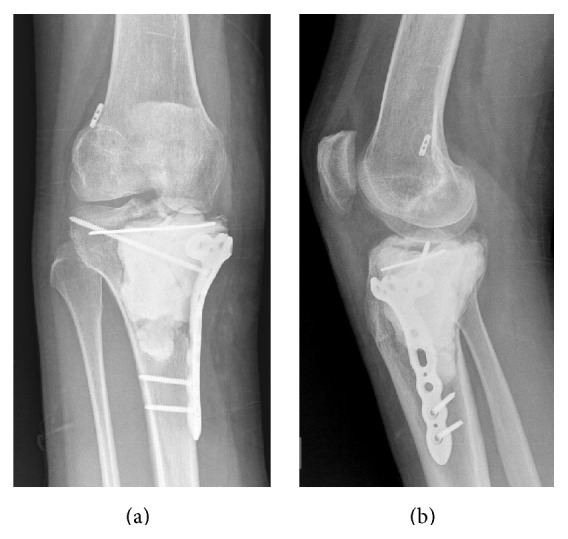
AP and lateral standing radiographs 4 months after giant cell tumor curettage and packing demonstrate stable postoperative change.

**Figure 11:**
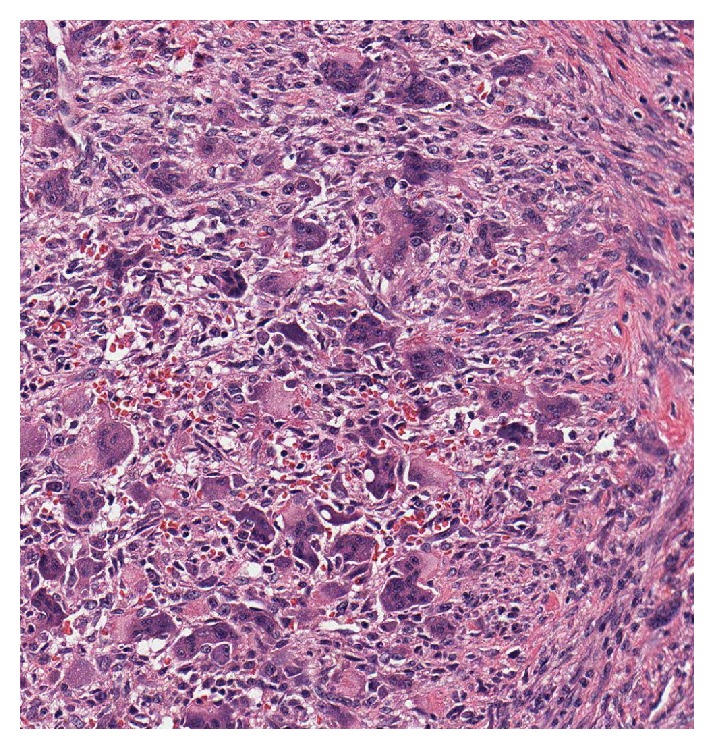
Hematoxylin and eosin stain histological slide.

Approximately 3 months after the giant tumor curettage and cementation, MRI and CT were obtained as a baseline. These studies demonstrated a new area of uncemented intramedullary cavity anterior to the cement (Figure 9). Given the short interval since prior MRI (Figure 7), findings are clinically thought to represent postoperative inflammatory change. Subsequent follow-up radiograph, which is approximately 4 months after the curettage and packing surgery, shows stable surgical change (Figure 10). The patient continues to show clinical improvement in her symptoms and continues with clinical and imaging surveillance.

## 3. Discussion

As the lesion progressed, it became apparent that the radiographic and MRI features of the lesion are suggestive of giant cell tumor of bone. Our case demonstrates serial imaging of histologically proven new development of giant cell tumor of bone within the previous ACL reconstruction surgical bed. To our knowledge, there have been two previous case reports of giant cell tumor which have occurred in the proximal tibia and distal femur around the fixation screws after bone-patella-bone anterior cruciate ligament reconstruction [1, 2].

The exact site of origin of GCT has been controversial. Murphey et al. suggest that GCTB likely arise from the metaphyseal side of the epiphyseal plate [3]. The present case also demonstrates new development and progression of the pathologically proven giant cell tumor consistent with this hypothesis.

World Health Organization (WHO) classifies giant cell tumor of bone (GCTB) as a benign, locally aggressive tumor. It is generally a benign, common bone tumor accounting for approximately 20% of benign osseous neoplasms, approximately 10% of primary osseous neoplasms, and 5% of all bone tumors [3, 4]. There is slight female gender predominance in its benign form, and it is typically seen between 20 and 40 years of age, with 80% of cases occurring between 20 and 50 years of age. The lesion is usually solitary. GCBT are most commonly seen about the knee, with the distal femur being more frequently involved than the proximal tibia. Clinically, affected patients often present with pain secondary to underlying bone destruction, which can predispose to pathologic fractures. Multifocal GCTB is rare, accounting for less than 1% of all cases. In 5–10% of cases, GCTB may be classified as malignant, which includes benign metastasizing giant cell tumor, sarcoma developing following radiation or other intervention of a preexisting giant cell tumor, primary transformation of preexisting giant cell tumor, and osteoclastic sarcoma.

Conventional radiographs often have classic findings and can be highly suggestive of the diagnosis of GCBT. These findings include eccentric, lytic lesion centered in the metaepiphysis extending up to the subchondral bone plate without internal mineralization in a patient with closed physis [3]. The margin of the lesion is typically nonsclerotic. Pereira et al. analyzed 30 patients diagnosed with giant cell tumor of bone for their MRI findings [4]. In this study, MRI features suggesting giant cell tumor typically included low T2 signal due to the fibrous component along with the deposition of hemosiderin within the tumor. Following the administration of intravenous contrast, typically there is heterogeneous enhancement pattern. Secondary aneurysmal bone cysts are relatively common within a GCT lesion of bone. Over 40% of cases had evidence of extraosseous involvement on MRI, among which four cases were radiographically occult.

Imaging differential diagnosis includes primary aneurysmal bone cyst (ABC) and chondroblastoma. Intravenous contrast administration on MRI is helpful in distinguishing GCTB with secondary ABC from primary ABC as the presence of enhancing soft tissue component is typically present in GCTB but not in primary ABC. Presence of extensive surrounding reactive edema within the marrow and soft tissues, sclerotic margin, and presence of chondroid matrix are helpful features distinguishing chondroblastoma from GCT. Additional differential diagnoses include metastasis, plasmacytoma, or multiple myeloma, which should be included based on patient's age, multifocality, and clinical history of known primary neoplasm. Telangiectatic osteosarcoma, giant cell-rich osteosarcoma, and fibroblastic osteosarcoma should be included in differential considerations when aggressive giant cell tumor of bone is suspected, as these subtypes of osteosarcoma do not produce osteoid matrix [5]. Furthermore, additional differential diagnosis should include clear cell chondrosarcoma and joint centric processes such as pigmented villonodular synovitis and geodes.

In the past, GCTB has been treated primarily by the means of surgical resection with curettage and placement of cement. Aggressive giant cell tumor may require wide excision and reconstruction utilizing modular endoprosthesis. Even with aggressive surgical treatment, the local recurrence rate was noted to range from 15 to 50% [4–6].
